# Women’s experiences of symptoms of posttraumatic stress disorder (PTSD) after traumatic childbirth: a review and critical appraisal

**DOI:** 10.1007/s00737-015-0560-x

**Published:** 2015-08-12

**Authors:** Stella James

**Affiliations:** PG. Cert. Professional Doctorate for Health and Social Care, London, Kent UK

**Keywords:** PTSD, Re-experiencing, Hot spots, Core beliefs, Dysfunctional assumptions, Negative automatic thoughts, Current threat, Rumination, Avoidance behaviour, CBT

## Abstract

This paper critically analyses nine studies on postnatal posttraumatic stress disorder (PTSD) following traumatic childbirth, in order to find common themes of PTSD symptoms, using the cognitive model of PTSD as a guide; it critically appraised one of the studies in depth and it attempted to explain the lived experience of women suffering from postnatal PTSD following traumatic childbirth and the suitability of cognitive behavioural therapy (CBT) for postnatal PTSD. This paper found that women following traumatic childbirth do experience postnatal PTSD; postnatal PTSD symptoms are similar to PTSD symptoms of other events and that CBT for PTSD of other events is just as effective for postnatal PTSD. Future recommendations include more qualitative studies with interpretative phenomenological approach in order to establish evidence-based CBT treatment for this client group, and more referrals need to be sent to the psychological services for CBT intervention.

## Introduction

Elhers and Clark (2000) suggest posttraumatic stress disorder (PTSD) to be a common reaction to traumatic events. The popular perception of PTSD is that it is a disorder that is mostly associated with traumatic events, other than traumatic childbirth. However, many women who have been through the experience of traumatic childbirth have expressed distressing symptoms of PTSD (Ayers et al. 2007; Stramrood et al. 2011a, b). Opportunities for this group of women’s PTSD symptoms to be treated appropriately and efficiently can be missed as they have not been defined as actual PTSD symptoms. If the PTSD symptoms can be established, then there might be more opportunities for this group of women to be treated in psychological therapy services with cognitive behavioural therapy (CBT) for PTSD symptoms. Thompson and Downe (2008) propose that despite the fact that professionals are becoming more aware of traumatic childbirth experiences and PTSD, not enough professional services are readily available. Therefore, the lived experience of this group of women needs to be established in order to provide appropriate CBT services.

For the vast majority of women, giving birth has been a satisfactory and rewarding experience (Nelson 2003); for others, it has been described as a distressing traumatic experience (Waldenstrom et al. 2004; Dahlen et al. 2010). Evidence shows that approximately 2 % of women fulfil diagnostic criteria for PTSD following childbirth (Ayers 2004; Olde et al. 2006).

Research evidence of women’s traumatic experience indicates experiences of intrusions, maladaptive beliefs, avoidance, re-experiencing, emotional numbing and arousal, hyperarousal, flashbacks and nightmares, dissociation, sense of threat, shame, anger and fear (Ayers et al. 2007). Other studies on women’s traumatic experience indicate extreme pain and inadequate pain relief during labour as associative factors (Allen 1998; Creedy et al. 2000); higher levels of obstetric intervention, i.e. assisted delivery using forceps or emergency caesarean section (Creedy et al. 2000; Soet et al. 2003); fear of death or physical harm to self or baby (Allen 1998; Beck 2004; Anderson and McGuiness 2008); and feelings of loss of control and powerlessness during labour (Ballard et al. 1995; Czarnocka and Slade 2000; Reynolds 1997).

Ayers et al. (2008) propose the need to establish whether the phenomenology of PTSD following childbirth is the same as PTSD after other events. This is important for appropriate and effective treatment for women following traumatic birth. They suggest the need to examine whether PTSD following childbirth is similar in terms of symptoms, course, duration, aetiology, effects and response to treatment. Beck et al. (2011) points out the importance of developing more appropriate services and providing effective treatment due to the burden of women’s postpartum physical and mental morbidity.

The quality of evidence for symptoms of PTSD following traumatic childbirth was substantially and collectively generated by all the nine papers reviewed in this paper. Studies indicate the persistence of PTSD symptoms following childbirth, and overall evidence also indicate typical symptoms of PTSD such as re-experiencing the trauma memories, perceived threat to self or baby, avoidance behavioural strategies, hyperarousal and dissociation (Ayers et al. 2007; Stramrood et al. 2012; Boorman et al. 2013).

A search for the review papers was conducted in October 2013 and reviewed between October and November 2013. Sixteen papers were originally read but were limited to nine papers written in English and published between 2007 and 2013. Searches for publications were made in Scopus, CINAHL, PsycInfo, Medline, PsycArticles databases and BABCP journals. Reference lists of some papers were also read to find additional and original relevant research. Search terms used were “traumatic childbirth”, “birth trauma”, “traumatic birth”, “PTSD and traumatic birth”. The terms were entered individually and also combined.

Included studies were studies that focused on women’s experiences of childbirth trauma, presentation of symptoms of PTSD and/or childbirth posttraumatic or postpartum stress symptoms following traumatic childbirth and CBT treatment of posttraumatic stress following childbirth and used the above terms in the title, abstract or key words. It was difficult to find purely qualitative studies (which would have addressed the subjective experiences of this group of women) based on the time span of within the last 5 years as requested, and so, both qualitative and quantitative studies within this period were used. Excluded studies focused mostly on pre-existing history of PTSD prepregnancy, postnatal depression, no experience of posttraumatic stress symptoms following childbirth and dated publications. There was an exception of one article that is 6 years old and is most relevant to this review which had to be included for in-depth critical appraisal (Ayers et al. 2007). This paper focuses on CBT for postnatal posttraumatic stress disorder. A summary of the nine papers chosen for review can be seen in Table 1 (Appendix 2).

## The review

In this review, the intention is to identify similar themes of PTSD symptoms in the findings of the reviewed papers and, in addition, to attempt to have answers to the following research questions:

- What is the lived experience of women who have had traumatic childbirth?
- Are the women experiencing actual symptoms of PTSD?
- Is CBT for PTSD as effective for posttraumatic stress symptoms following childbirth as it is for other causes of PTSD?

## Themes

Four common themes (Table 2: Appendix 2) were developed during the review of the nine papers. All nine papers in the review were in agreement that PTSD following traumatic childbirth does occur.

The themes are as follows:

- Nature of trauma memory
- Negative appraisal of trauma and/or its sequelae
- Current threat
- Strategies intended to control threat/symptoms

**Fig. 1:**
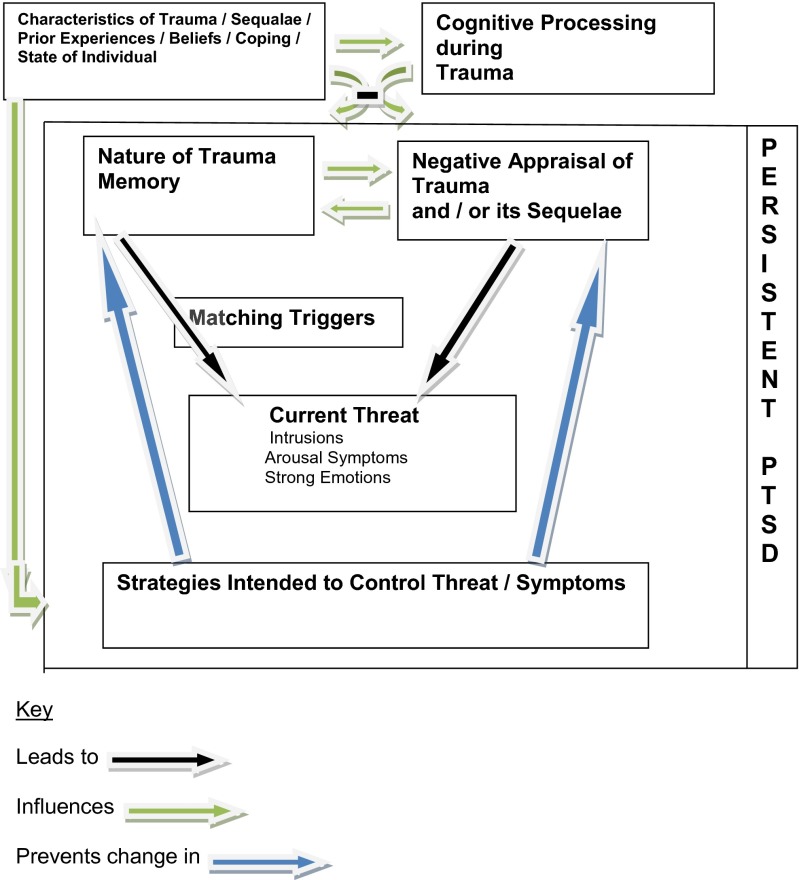
Cognitive model of PTSD (Elhers and Clark 2000)

The themes are guided by the cognitive model of PTSD (Elhers and Clark 2000). This model explains the link between the themes which proposes that the characteristics of the trauma and cognitive processes during trauma influence the nature of trauma memory and the negative appraisals of the trauma and or its sequelae (which also influence each other). It proposes that both the nature of trauma memory and the negative appraisal of trauma and its sequelae lead to the current threats, which contribute towards strategies intended to control threats/symptoms, which in turn prevent the change of the trauma memory and the negative appraisal (which maintains the problem). The model proposes that the matching triggers are reminders of the trauma memory (Fig. 1: Appendix 2).

### Nature of trauma memory

Researchers report re-experiencing as one of the most frequently experienced symptoms of PTSD in traumatic childbirth (Ayers et al. 2009). They report women with PTSD following traumatic childbirth sharing similar symptoms, such as re-experiencing with those observed with PTSD following other traumas (Ayers et al. 2008). A case study reports a woman reliving the birth experience (flashback) during a therapy session, which includes seeing herself lying in the delivery room (Ayers et al. 2007). Another woman is described experiencing intense distress at exposure to internal or external cues that resembled an aspect of the delivery (Stramrood et al. 2012). Zimmerman (2013) proposes that the experience of caesarean section, the use of forceps or vacuum device, vaginal examination, nakedness in the presence of others, pelvic pain, and perceived sense of lack of control during birth also contribute to re-experiencing.

### Negative appraisal of trauma and/or its sequelae

Women expressed negative appraisal of the experience leading to the traumatic childbirth, the process during the traumatic childbirth and the experience following the traumatic childbirth (Ayers et al. 2007; Boorman et al. 2013; Zimmerman 2013).

The women expressed negative automatic thoughts, dysfunctional assumptions and core beliefs about themselves, others and the world around them (Beck et al. 1979). For example, case 1 in the study of Ayers et al. (2007) reported hot spot (Grey et al. 2002; Nijdam et al. 2013) as she thought she might die. Her negative automatic thoughts included thoughts such as “I can’t cope and they don’t want to know”. Her dysfunctional assumptions included, “if I am upset, they will think I am weak”. Her core beliefs included “I am a failure” and “they are rejecting”. This is in accordance with a study of Dekel et al. (2013) that examined the bi-directional relationship between PTSD and posttrauma cognitions in the long term following trauma. They found that PTSD symptoms predicted subsequent relatively negative cognitions concerning the self and the world. They propose that not only does negative cognitions trigger the development of PTSD and its maintenance (Foa and Feeny 2006) but PTSD symptoms may also contribute to the deterioration of negative cognitions and keep them negatively enduring.

Researchers report women’s “shattered assumptions” are associated with care and beliefs (Ayers et al. 2008); women’s beliefs of the traumatic childbirth as having lack of control over the decisions made during the traumatic childbirth (Boorman et al. 2013); their cognitive attention/process is focused more on threat words, when their cognitive attention bias with reaction time is tested (Dale-Hewitt et al. 2012). This is in accordance with Elhers and Clark’s (2000) proposal of cognitive processing during trauma, which leads to the perception of current threat. Researchers (Leeds and Hargreaves 2008; Thompson and Downe 2008) reported hot spots of “threatened death, injury and threat to physical self, fear for baby and unexpectedness of procedures”. Stramrood et al. (2012) report women’s appraisal of the childbirth as “violent and abusive” and the imbalance of power. In addition, Zimmerman (2013) reports the women’s appraisal of traumatic childbirth as high expectation of self during childbirth, future fear of birth, further blame of child for emotions and pain, and therefore, having thoughts of harming the child or overwhelming thoughts of need to protect the child.

### Current threat

Researchers report a common theme of women’s description of current threat as emotional numbing and hyperarousal, anxiety, panic, depression, suicidal, dissociation of the mind and body, vulnerability, confusion, helplessness, low self-esteem, intense burst of anger, shame, fear, disturbed sleep, feeling threatened, stress and feeling disconnected. This supports the proposal of the cognitive model of PTSD (Elhers and Clark 2000) which proposes that both the nature of trauma memory and the negative appraisal of trauma and its sequelae (as can be seen in above themes) are contributing factors to the current threats. For example, they propose a sense of threat as a “sense of impending doom” (Elhers and Clark 2000, pg. 344), which leads to anticipatory anxiety and a sense of worse to come.

### Strategies intended to control threat/symptoms

Women describe their common strategies as avoidance of triggers of trauma memory, i.e. hospital, words associated with labour (Dale-Hewitt et al. 2012); they described putting barriers up, avoiding being judged and withdrawal from social activities. Researchers report avoidance of baby, sex, future pregnancy and overprotection of the baby (Strong 2012, cited by Zimmerman 2013). Ruiter and Bosschot (1994) in Dale-Hewitt et al. (2012) report the “strategic cognitive avoidance” of attention bias away from labour words. This supports the proposal of Elhers and Clark (2000) who propose that current threats contribute towards strategies intended to control threats/symptoms, which in turn prevents the change of the trauma memory and the negative appraisal (which maintains the problem).

## Discussion

In order to answer the research questions in this review, the qualitative approach appears to be the most suitable approach to show that traumatic childbirth is leading to PTSD. The reason for the suitability of this approach is that the method of qualitative data collection (in-depth interview) is able to establish factors about the subjective experience of the women, the structure of the interview is sensitive and flexible, and therefore, actual lived experiences can be generated and it enables the interviewee more control in expressing themselves (Graham 1984). In support, Nicholls (2009a, b) proposed that interviews in qualitative research studies emphasize the need to understand the lived experience of health and illness. This is important, as applying the in-depth interviews to studying women with postnatal PTSD (PNPTSD) contributes towards clinical practice, it is beneficial and it enables researchers’ awareness of the presentation of PNPTSD in a clinical setting. The approach enables inductive reasoning which allows for the establishment of a theoretical understanding (Carpenter and Suto 2008) of PNPTSD as a result of exploration.

Research indicates that qualitative research focuses on samples that provide appropriate and adequate insight into the person’s experience of the world, it focuses on depths and richness to explanations of people’s experience (Nicholls 2009a, b); sampling in qualitative research explores diversity, difference, variations and heterogeneity (Morse 1991).

The qualitative study of Thompson and Downe (2008) addressed the first question of the lived experience of the women because it adopted an interpretative phenomenological approach for the study and therefore shows that it is addressing their lived experience. The study collected data through in-depth interviews, and an interpretive analytic approach was applied to elicit deep meanings of the lived experiences. This approach is also important in answering this question because not only did it elicit the cognitive appraisal of trauma events but it was also able to elicit hot spots which are vital in the cognitive processes in PTSD of other events. In-depth interview is suggested to guide conversations (Lofland and Lofland 1995); it is useful for “testimony studies” when used with evaluations of service (St. Leger et al. 1992) and for life or oral histories. This is important as it also enhances the researcher’s understanding of the individual, of social life and of social interaction (Atkinson 1998). A possible limitation of this study though is the variation in the interval between the traumatic childbirth and the interviews, which varied between 15 months and 19 years. The trauma memory or flashback still being experienced by the women with 15 months gap might be more prominent than the women with a 19-year gap.

Zimmerman (2013) also answered the three research questions for this review. It proposes that proper diagnosis and treatment of PTSD are vital for the mother and her relationship with her family and baby. This study helps towards answering the question of the importance of the need to apply CBT intervention to this group of women by indicating psychological and psychosocial treatment as effective intervention for improving these women’s quality of life. It indicates the necessity of cognitive complementary treatment and emphasizes that medication alone is not enough. It proposes CBT treatment for maladaptive perceptions, emotional management adaptability and relaxation techniques. It proposes family therapy (as family members are also affected: Ayers et al. 2006) and marriage counselling; it proposes EMDR to determine positive memory and hypnotherapy (Lynn and Kirsch 2006); as well as it proposes CBT with hypnotherapy (Bryant et al. 2005).

Leeds and Hargreaves (2008) approached their study quantitatively and were able to elicit all four themes as shown in Table 2, Appendix 2, as well as demonstrated that a significant number of women continue to experience some level of PTSD and depression at 9.5 months following traumatic childbirth. Compared to the previous studies (Czarnocka and Slade 2000; Soet et al. 2003) which have reported women continuing to experience the symptoms of PTSD in the first month after childbirth, this is the first study that ascertained that women (23.5 %) continue to experience some level of PTSD at 9.5 months following childbirth. This could be down to the type of questionnaires used which related to childbirth: Perinatal Posttraumatic Stress Disorder Questionnaire (PPQ) (Quinnell and Hynan 1999) which is not subjective enough to cover and treat actual lived experience; Posttraumatic Stress Disorder Checklist (PCL) (Weathers et al. 1994) which is specific to re-experiencing, avoidance and increased arousal in DSM-IV (APA 1994) which also prompts distress levels. In addition, the study included the Perception of Labour Questionnaire (Czarnocka and Slade 2000) which asks participants about their perceptions of their experience of labour and delivery. This is a questionnaire that was not found in other quantitative studies reviewed.

The elicited themes were as follows:

- Nature of trauma memory
- Negative appraisal of trauma and or its sequelae
- Current threat
- Strategies intended to control threat/symptoms

The links between the themes show that where women with PNPTSD have experienced traumatic childbirth and had fearful traumatic thoughts during the trauma, this influences the nature of their memory of the trauma (flashback and re-experience) and how they negatively interpret the traumatic childbirth and its consequences, which then lead to, for example hyperarousal, panic and emotional numbing (current threats). This then contributes towards the women developing strategies to control perceived threats by avoiding any triggers of trauma memory, which in turn prevents the change or reduction of trauma memory and negative perceptions, which continues to maintain the problem. This fits in with the CBT model of PTSD (Elhers and Clark 2000) earlier explained.

## In-depth critical appraisal of Ayers et al. (2007)

Initial reasons for choosing this paper for critical appraisal are its originality which constitutes important knowledge. It establishes new information for clinical practice in cognitive behavioural therapy. It answers the three research questions, and it is most relevant for future clinical practice of CBT treatment for PNPTSD. The methodology used was a qualitative approach to case study which is appropriate in generating an understanding of the women’s actual lived experience of their traumatic childbirth. Its in-depth interviews established the richness of their unique and subjective experience. The study was able to elicit themes which fit the CBT model of PTSD for other events and apply the CBT model of PTSD to their assessment and treatment. The study’s inductive reasoning has enabled the establishment of a theoretical understanding of the applicability of CBT for PTSD of other events to PNPTSD. According to (Finlay 2009), in research, it is important to focus on what the participants are experiencing and how, and the relationship between the participant and the meanings of the things that they are focusing on and experiencing. This is exactly what the Ayers et al.’s (2007) study focused on as recommended by Finlay (2009).

Although suitable participants were chosen, the process in which they were recruited is not clear nor is the representativeness of this sample. Inclusion and exclusion criteria or possible dropouts are not clear in this study, and the characteristics of those not included in this study also not evident. The process of consent or if confidentiality approval was explained to the participants was not explicit in this study. The process of recruitment needs to be made clear. For example, the National Research Ethics Service (NRES) (2008) states that before consent, researchers have to provide information for the participant. It was not clear if it occurred.

It was clear that they were clinically assessed for treatment, but the process and amount of assessment were not explicit; who assessed them; nor the process of how they were interviewed before the assessment. Details of experiences pre, during and post childbirth were comprehensively described, as was the precipitating and perpetuating factors of the trauma experienced. Their individualised formulation diagrams were helpful in understanding their cognitive processes. Treatment was explained; however, in case 1, how many times the reliving was repeated was not explicit. The exact techniques of cognitive restricting applied would have been useful for another researcher attempting to replicate this study. Although it must be emphasised that CBT is a tailor-made intervention applied according to individual needs, it would have been useful for future research to understand better how the anger and low self-esteem were reduced (i.e. which CBT intervention applied). Also, it was not clear if the visits to the labour ward were graded exposure or a one off.

The discussion of the study was not linked back to the literature review at the beginning of this paper. Linking the discussion of the study back to literature review at the beginning of a paper has been found to provide opportunity to revisit the knowledge and ideas, including strengths and weaknesses that have already been reviewed. Rather, other literature was linked to the discussion. The researchers chose appropriate aims of the study. For example, they pointed out that the case studies illustrate the use of formulation as central to CBT. Grant et al. (2008) propose that the key to effective therapeutic interventions in CBT is the development of a therapeutic relationship, a detailed and systematic assessment. Other suggestins are that a formulation is to guide practice, and treatment to be tailored to individual needs (NICE 2012), the role of beliefs and appraisal is central in PNPTSD (Janoff-Bulman 1992).This is important because in developing a therapeutic relationship, it can be helpful to develop a theoretic model of the origins of their problems with the participant (Leahy 2008).

The researchers highlighted factors relevant to health professionals involved in the management of pregnancy and birth. Highlights included influence of previous events and beliefs on the women’s perceptions and emotional response to pregnancy, birth and expectations of birth, sensitivity to management of events during birth impacting on the woman’s experience and appraisal of events, the need to be more sensitive to vulnerability during traumatic childbirth, issues around recognising and diagnosing both PNPTSD and postnatal depression (Kessler et al. 1995) and issues around women being avoidant of reminders of trauma memories of the birth (hospitals, health professionals due to loss of confidence in them and due to feeling judged or not taken seriously). The case studies also demonstrate the effective treatment of CBT for PNPTSD. This study, however, was limited to only two cases. Other possible forms of study that could create evidence-based treatment in this are comparisons of two types of CBT treatment, for example standard exposure in vivo (Elhers and Clark 2000), compared to imagery work (Hackmann et al. 2011) to treat their PTSD symptoms.

Their conclusion accurately reflected on findings and can be linked to the title and the aims. The researchers feel that their increase of PNPTSD awareness will inform the diagnosis and treatment of women with this presentation and inform the management of pregnancy, birth and clinical practice. This study is beneficial and influential to CBT practice as it informs the clinical practice. This study confirms that this client group is truly experiencing PTSD symptoms following traumatic childbirth and needs to be cared for using the cognitive model of PTSD to formulate and treat their symptoms.

## Conclusion

The findings of this review and the in-depth critical appraisal of Ayers et al.’s (2007) paper, all together, show that the studies were able to establish four main themes of PTSD from women who have experienced traumatic childbirth, which fits neatly into the CBT model of PTSD (Elhers and Clark 2000). The findings indicate that women who have gone through traumatic childbirth experience the same PTSD symptoms and those of PTSD of other traumatic events. These experiences include trauma memories (Elhers and Steil 1995; van der Kolk and Fisler 1995), negative appraisal of trauma and its sequelae (Foa and Rothbaum 1998; Lee et al. 2001) and avoidance and safety behaviour (Salkovkis 1996).

This means that the CBT model of PTSD used for other trauma events can be applied to women PNPTSD. This model could be beneficial, a good implication for clinical practice as well as a new innovation in psychological treatment for this group of women.

Although many questionnaire approaches used in some of the studies may have elicited PTSD symptoms experienced, it does not necessarily always fully address cognitive processes such as the hot spot as well as ruminations.

The recommendations of this review are as follows:

- To carry out evidence-based treatment of CBT for PTSD (includes anxiety management, reliving of which imagery and in vivo exposure, and cognitive restructuring), for PNPTSD. This may confirm the suitability of CBT for PNPTSD and generate a new theory.
- To carry out a randomized controlled trial of treatment, comparing of two types of CBT treatment, for example standard exposure in vivo (Elhers and Clark 2000), compared to imagery exposure (Hackmann et al. 2011) to treat PNPTSD. Each treatment will include anxiety management and cognitive restructuring, respectively. This might generate evidence that imagery exposure alone might be enough PNPTSD.
- To carry of qualitative studies (with higher numbers of participants) with interpretative phenomenological approach, with emphasis on meta-cognitions and ruminations. More emphasis is needed on the process of in-depth interview as opposed to questionnaires.

This review suggests consideration for, training of CBT therapists in PNPTSD, referral of this client group to CBT service and psychoeducational information of PTSD provided to this group of women which empowers them.

The reviewed papers have been able to establish that women who have gone through traumatic childbirth do experience actual symptoms of PTSD. It has also established that CBT for PTSD of other events is just as effective for PTSD following traumatic childbirth. Therefore, CBT should be an option used as an intervention for women with PNPTSD.

## Appendices

### Appendix 1. Defined terms

*Traumatic childbirth*: Beck and Watson (2008) defined this as “actual or threatened injury or death to the mother or her baby”. Some examples of traumatic childbirth can be suggested to be an unexpected or emergency caesarean section, painful internal examination, instrumental delivery (forceps), episiotomy, tear, unsympathetic health professionals, feeling vulnerable out of control etc.

*Posttraumatic stress disorder*: According to Elher’s and Clark’s cognitive model, PTSD persists when past trauma is processed in a way that leads to a sense of serious threat. “The sense of threat arises as a consequence of (1) excessively negative appraisals of the trauma and/or its sequelae and (2) a disturbance of autobiographical memory characterized by poor elaboration and contextualization, strong associative memory and strong perceptual priming. Change in the negative appraisals and the trauma memory are prevented by a series of problematic behavioural and cognitive strategies” (Elhers and Clark 2000). This is supported by other psychological models of PTSD within the cognitive framework (Brewin et al. 1996; Foa and Rothbaum 1998; Litz and Keane 1989). DSM-IV (American Psychiatric Association: APA 1994) suggests that PTSD occurs following traumatic events in which individuals experience a threat to their own life or the lives of others or a threat to their own or others’ physical integrity.

*Hot spots*: This has been defined as intrusions that are mapped by the patient or client as the worst moments of the trauma (Grey et al. 2002). Hot spots have also been shown to be accompanied by a range of emotions: anger, sadness, fear, shame, guilt, helplessness and horror (Holmes et al. 2005).

*Re-experiencing*: This has been described as a core symptom of PTSD (Elhers et al. 2004). This includes flashback, intrusive images, nightmares, and distress and psychological reaction when confronted by reminders (APA 1994).

*Core beliefs*: This gives rise to beliefs people have about themselves, others, the world and the future. Typical statements about the self that are an absolute, global and stable nature (i.e. “I am a failure”, “I am useless”) formed on the basis of early experiences. Typical statements for others are “people will judge me”; example of the world is “this world is a dangerous place” and example of the future can be “there is no hope for the future” (Beck et al. 1979; Clark and Beck 1999; Padesky and Greenberger 1995).

*Dysfunctional assumptions*: These have been reported to be highly individualised, conditional and generalised rules, i.e. “if I am nice to everyone, I will be liked”, “I must be a perfect mother, if not then I am a failure” (Bennett-Levy et al. 2004; Padesky and Greenberger 1995).

*Negative automatic thoughts (NAT’s)*: These are unhelpful thoughts that are associated with strong emotions and, as a consequence, produce associated behaviour. Typical NAT’s are “I am always unlucky”, “I never get things right” (Crandell and Chambless 1986; Brewin 1989; Barber and Derubeis 1989; Padesky and Greenberger 1995).

*Rumination*: This has been defined as “behaviour and thoughts that focus one’s attention on one’s depressive symptoms and on implications of these symptoms” (Nolen-Hoeksema 1991).

*Cognitive behavioural therapy*: CBT is structured and time limited and focuses on the here and now issues and helps people understand the links between thoughts, feelings and behaviour. The therapist and the patient work together to identify the thoughts, attitudes and beliefs that are associated with particular problems and to discover if there are more realistic and helpful ways of seeing things. With the support of the therapist, patients are then encouraged to try out new ways of dealing with problems and to evaluate what works best for them. The cognition focuses on strategies to modify the core beliefs, dysfunctional assumptions and negative automatic thoughts, while the behaviour focuses on maladaptive behaviours and emotions which include rumination (Beck 1967; Beck et al. 1979).

### Appendix 2

**Table 1:** Studies of women’s experiences of posttraumatic symptoms after traumatic childbirth

Author/location	Methodology/approach	Method	Aim(s)	Sample	Time of recruitment	Inclusive /exclusive criteria
Ayers et al. (2009), UK	Longitudinal (community) and cross-sectional (Internet) study	Questionnaires PDS: 1997 version (self report) and EPDS completed in pregnancy, 3 and 12 months after birth	Examine presentation and symptom structure of PTSD after birth, risk factors for PTSD after birth and risk factors for disability/impairment after birth	1423 women recruited (502 via Community and 921 via internet) Age not specified	Last trimester of pregnancy	Women from Internet and community. Exclusion not clarified
Ayers et al. (2008), UK	Qualitative	A report of content of 4 discussions on prevalence and comorbidity, screening and treatment, diagnostic and conceptual issues, and theoretical issues	To summarize current understanding of PTSD following childbirth, to identify conceptual and methodological issues and to offer recommendations for future research	Women with PTSD after giving birth (globally)	Not specified	Not specified
Ayers et al. (2007), UK	Qualitative Case study	Application of cognitive behavioural therapy intervention for postnatal PTSD for each participant	To describe the use of CBT interventions for postnatal distress and illustrate common themes in postnatal PTSD. To emphasize the importance of diathesis-stress understanding of postnatal PTSD	2 women with postnatal PTSD (a 35 years old with 14 month old child and a 32 years old with 8 month old child)	Actual time of recruitment was not specified	Criteria not specified
Boorman et al. (2013), Australia	Quantitative (part of a larger RCT)	Questionnaires completed at 3rd trimester of pregnancy and 14 days post partum. EPDS and DASS-21 both completed at time 1 and 2	To differentiate criterion A1 (threat) and A2 (intense emotional response) for PTSD of DSM-IV, to explore their individual relationship to prevalence rates for posttraumatic stress, each other and associated factors to childbirth trauma	1040 Women in their third trimester of pregnancy attending antenatal clinic appointment	3rd trimester of pregnancy	Inclusion: Above 17 years old, expected to give birth to live infant and able to complete questionnaires in English. Exclusion: those undertaking psychological treatment and those who did not complete antenatal questionnaire
Dale-Hewitt et al. (2012), UK	Quantitative	Experimental design Stroop task (Stroop word list) Questionnaires: IES, PTSD-Q, the experience of birth scale and the Edinburgh postnatal depression scale	Investigate primarily whether PTS symptoms after childbirth are associated with attention bias to labour-related words. To explore whether such attention biases are related to experience of trauma, subjectively to with or without posttraumatic responses	50 women, who experienced labour and delivery as stressful, responded with fear, helplessness and horror	Between 6 weeks and 6 months of given birth	Inclusion: Above 16 years old, with children with no health problems nor require special care for more than 48 h Exclusion: those with selective caesarean section, not fluent in English and those who were blind
Leeds and Hargreaves (2008), UK	Quantitative Retrospective, self-reported study	Questionnaires PPQ, PCL, EPDS, Perception of Labour and Delivery Questionnaire	Address prevalence for PTSD after childbirth, address recovery rate in the longer term, investigate whether women have experienced birth-related trauma at 6–12 months, establish how many women will continue to experience partial PTSD, determine how many women report PTSD after child birth, how many women are affected by postnatal depression and determine which factors might affect levels of psychopathology in women 6–12 months after child birth	1000 women identified but 500 women selected. Women in North Wales who had given birth to a baby between October 2003 and March 2004	6–12 months postpartum	Inclusion: as previously mentioned Exclusion: Women with stillbirth or subsequent death of baby, severe maternal mental health problems (psychosis, severe postnatal depression), women who might become distressed as mentioned by their GP and families who had experienced maternal death
Stramrood et al. (2012), Netherlands	Qualitative Case study	Application of eye movement desensitization and reprocessing (EMDR) treatment for women with symptoms of PTSD following childbirth Individual clinical interviews	To evaluate the possibility of using EMDR treatment for women with symptoms of PTSD following childbirth To reduce posttraumatic stress symptoms, to increase confidence and to create positive perception of upcoming delivery	3 women with posttraumatic stress following childbirth and who are also with their second pregnancy Ages 29, 21 and 25 years	During their second pregnancy; actual time was not specified	Criteria not specified
Thompson and Downe (2008), UK	Qualitativeinterpretative phenomenology	In-depth interviews	To explore the lived experience of, and personal meanings attributed to, a traumatic birth	14 women Ages 27–40 years	Interview for the study varied between 15 mn and 19 years posttraumatic birth	Inclusion: phase 1, participants who had experienced both a self-defined traumatic and positive birth. Phase 2, women who had a traumatic birth and were pregnant with a further child
Zimmerman (2013), US	Qualitative	Studies information gathering of evidence base	To examine PTSD as relates to a mental disorder after childbirth, its prevalence among women, causes and risk factors, symptoms, diagnosis and treatment	Studies on women with PTSD symptoms experienced after childbirth Age not specified	Not specified	Not specified

**Table 2:** Common themes

	Nature of trauma memory	Negative appraisal of trauma and or its sequelae	Current threat	Strategies intended to control threat/symptoms
Ayers et al. (2009)	Re-experiencing of trauma (most frequently experienced)	Hot spot (thought she might die) “I can’t cope” “If I am upset, they will think I am weak” “I am a failure”	Hyperarousal and numbing	Avoidance of triggers
Ayers et al. (2008)	Re-experiencing of trauma memory		Hyperarousal	Avoidance of triggers
Ayers et al. (2007)	Re-experiencing of trauma memory. Flashbacks, nightmares and physiological and emotional reactions to trauma triggers		Dissociation, panic, depressed, vulnerable, shame, anger, emotional numbing, disturbed sleep	Avoiding being judged, internalising feelings, avoidance of triggers
Boorman et al. (2013)		Belief of lack of control over decisions made	Sense of threat, fear, anxiety, depression, stress	
Dale-Hewitt et al. (2012)		Biased cognitive attention/process focused more on threat words (labour words)	Sense of threat, hyperarousal	Strategic cognitive avoidance of labour words perceived as threat
Leeds and Hargreaves (2008)	Re-experiencing of trauma memory	Hot spots of, “threatened death, injury and threat to physical self, fear for baby	Distress, hyperarousal, depression	Avoidance of triggers
Stramrood et al. (2012)	Re-experiencing of trauma memory (response to internal/external cues)	Belief of trauma as, “one big drama”, being “bruised”; “fear of going crazy if emotions are no put away”, “staff did not take me seriously”	Hyperarousal, numbing	Avoidance of triggers
Thompson and Downe (2008)		Hot spot, “death was imminent” Appraisal of childbirth as, “violent and abusive”	“Being disconnected”, sense of dissociation from birth, dissociation of mind and body	
Zimmerman (2013)	Re-experiencing of trauma memory	Low view of self, blame of baby’s father, thoughts of harming or protecting the baby, high expectation of self during birth, future fear of sex and or getting pregnant	Extreme depression, suicidal, anger, anxiety, low self-worth	Avoidance of sex and future pregnancy, avoidant or over protectiveness of baby
